# The Association of Insulin Sensitivity, Secretion, and Clearance With Subclinical Atherosclerosis in Middle‐Aged Adults Without Diabetes: A Cross‐Sectional Analysis of the SCAPIS Cohort

**DOI:** 10.1111/1753-0407.70161

**Published:** 2025-10-19

**Authors:** Rebecka Renklint, Per Liv, Ioannis Katsoularis, Tommy Olsson, Julia Otten

**Affiliations:** ^1^ Department of Public Health and Clinical Medicine Umeå University Umeå Sweden

## Abstract

**Object:**

Type 2 diabetes (T2D) is an established risk factor for cardiovascular disease (CVD), with increased risk already observed in the prediabetic state. This study aimed to investigate the association of insulin sensitivity, secretion, and clearance with subclinical atherosclerosis in a randomly selected cohort of Swedish adults aged 50–64 years without known diabetes.

**Material and Methods:**

For this cross‐sectional analysis, data from the Umeå site of the Swedish CArdioPulmonary BioImage Study (SCAPIS) were used, which included 2507 individuals aged 50–64 years. After applying exclusion criteria, 2054 participants remained. Insulin sensitivity, secretion, and clearance were calculated using an oral glucose tolerance test (OGTT). Atherosclerosis was assessed by coronary computed tomography angiography (CCTA) and carotid ultrasound, yielding coronary artery calcification scores (CACS), coronary segment involvement scores (SIS), and total carotid plaque counts. Ordinal regression models analyzed associations between insulin measures and atherosclerosis, adjusting for cardiovascular risk factors.

**Results:**

Lower insulin sensitivity, as measured by the GUTT index, was associated with higher CACS and SIS, but not with carotid plaque count. No significant relationship was found between insulin secretion (Insulinogenic Index) and any atherosclerotic marker. Reduced insulin clearance was associated with CACS and SIS in unadjusted analyses; however, these associations did not persist after multivariable adjustment.

**Conclusion:**

In individuals without diabetes, insulin resistance is associated with markers of subclinical coronary atherosclerosis, reinforcing its role in early CVD. Insulin secretion or clearance are not directly associated with measures of subclinical atherosclerosis in this population but may contribute indirectly via effects on circulating insulin levels.

## Graphical abstract

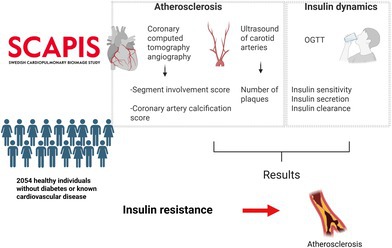

**Summary**

- We studied 2054 Swedish adults (aged 50–64) without diabetes from the SCAPIS cohort and found that lower insulin sensitivity was associated with early signs of coronary atherosclerosis, measured by segment involvement score and coronary artery calcification score.
- No significant associations were observed for insulin secretion or clearance after adjusting for cardiovascular risk factors.
- We conclude that insulin resistance is linked to early coronary atherosclerosis and should be addressed at an early stage.

## 1 Introduction

Type 2 diabetes (T2D) is a well‐established risk factor for cardiovascular disease (CVD), and even prediabetes promotes the development of atherosclerosis [1]. T2D is characterized by decreased insulin sensitivity, that is, increased insulin resistance, and a compensatory increase in insulin secretion by the pancreatic beta cells to maintain normoglycemia. Before overt hyperglycemia occurs, hyperinsulinemia may persist for several years to maintain normoglycemia. There is growing evidence that chronic hyperinsulinemia itself promotes insulin resistance and increases CVD risk [2, 3].

In early prediabetes, insulin resistance leads to an increase in very low‐density lipoprotein (VLDL) particles, which, when metabolized, promote atherogenesis [4]. Insulin resistance also leads to pro‐inflammatory and pro‐thrombotic states that further accelerate atherogenesis [5]. To compensate, beta cells increase insulin secretion, and the resulting hyperinsulinemia increases low‐density lipoprotein (LDL) synthesis, vascular smooth muscle cell proliferation, and activates inflammatory processes [6]. Compared to individuals with prediabetes and lower insulin secretion, individuals with high insulin resistance and compensatory high insulin secretion have a lower risk of developing diabetes, while the risk of all‐cause mortality and diabetes complications is increased [7].

In addition to insulin sensitivity and secretion, insulin clearance plays a crucial role in the regulation of circulating insulin levels, determining the variability of fasting insulin [8]. Reduced insulin clearance and the resulting hyperinsulinemia have been found to increase the risk of atherosclerosis, independent of insulin resistance [9]. Reduced insulin clearance is most commonly driven by the accumulation of intrahepatic lipids [10], but has also been associated with aging, ethnicity, obesity, and polycystic ovary syndrome [11].

Given the increased cardiovascular risk associated with prediabetes, early detection of atherosclerosis is crucial. The coronary artery calcification score (CACS), measured by non‐contrast computed tomography (CT), is widely used to assess coronary calcification and improves the prediction of cardiovascular risk beyond traditional risk assessment tools [12]. In addition, carotid artery plaque burden independently predicts multivessel disease of coronary arteries and future CVD risk [13].

In this study we examined the associations of insulin sensitivity, secretion, and clearance with subclinical atherosclerosis in a randomly selected population aged 50–64 years without diabetes. We hypothesized that insulin resistance, secretion, and clearance would each be associated with subclinical atherosclerosis.

## 2 Method and Material

### 2.1 Study Cohort

The Swedish CardioPulmonary BioImage Study (SCAPIS) is a multicenter, population‐based observational study conducted at six different sites in Sweden. The aim is to improve the prediction and prevention of CVD and chronic obstructive pulmonary disease (COPD). Men and women aged 50–64 years were randomly recruited between 2014 and 2018, and data were collected using questionnaires, anthropometry, blood pressure measurement, blood sampling, pulmonary imaging, and comprehensive cardiovascular imaging according to a standardized SCAPIS protocol, which has been described in detail elsewhere [14]. For the present study, only data from the Umeå site (*n* = 2507) were used, as the oral glucose tolerance test (OGTT) was only performed at this site. Participants were excluded if they had known diabetes; were newly diagnosed with diabetes during the study based on fasting glucose level, 120‐min glucose level, or HbA1c level; had clinically manifest atherosclerotic disease, defined as a self‐reported previous acute myocardial infarction (AMI), stroke, coronary artery bypass grafting (CABG), or percutaneous coronary intervention (PCI); did not consent to the storage of biobank samples; or did not provide blood samples for subsequent insulin analysis. The study was approved by the Ethics Committee of Umeå University, and written informed consent was given by all participants.

### 2.2 Study Procedure

Systolic and diastolic blood pressure was measured using an automatic device (Omron M10‐IT, Omron Health Care Co, Kyoto, Japan), and participants self‐reported whether they had taken antihypertensive medication in the previous two weeks. Fasting plasma cholesterol levels were analyzed with the CHOL2 (2600) (Roche Diagnostics Scandinavia AB); high‐density lipoprotein (HDL) levels with the HDLC4, 700 test (Roche Diagnostics Scandinavia AB); and triglycerides with the TRIGL 1000 test (Roche Diagnostics Scandinavia AB). HbA1c was determined from a fasting venous blood sample using the Tosoh HLC G11 analyzer (Tosoh, Japan).

### 2.3 OGTT

After an overnight fast, a venous blood sample was taken and capillary glucose levels were measured using Hemocue glucose 201 RT (HemocueAB, Sweden) to determine baseline glucose (glucose 0). When fasting capillary glucose measurements were not available, fasting venous glucose measurements were used instead, analyzed with the GLUc3 assay on the Cobas Pro system (Roche Diagnostics Scandinavia AB). After the fasting measurements, participants received an oral solution containing 75 g glucose. After 30 and 120 min, capillary glucose was measured (glucose 30 and glucose 120 respectively), and additional venous blood samples were collected. The venous blood samples were centrifuged and stored at −80°C until further analysis. Insulin concentrations were measured from venous blood after 0, 30, and 120 min (insulin0, insulin30, insulin120), and C‐peptide levels were measured from fasting samples (C‐peptide0). Insulin and C‐peptide were analyzed using the Elecsys assay (Roche Diagnostics Scandinavia AB).

### 2.4 Imaging and Image Analysis

The details of cardiac imaging including acquisition, processing, analysis, reconstruction, reading, and interpretation have been described in detail elsewhere [15]. In brief, coronary computed tomography angiography (CCTA) data were acquired using a multi‐slice CT scanner (Siemens, Somatom Definition Flash, Siemens Healthineers, Erlangen, Germany). In preparation for CCTA, participants were given a beta‐blocker to control heart rate and sublingual glycerol nitrate to dilate the coronary arteries. The contrast agent iohexol (GE Healthcare, 350 mg l/ml) was administered at a dose of 325 mg l/kg body weight and CCTA was performed at a voltage of 100–120 kV [15]. Coronary artery calcification (CAC) images were obtained using electrocardiogram‐guided, non‐contrast CT scans performed at 120 kV. All CCTA images were visually inspected for signs of atherosclerosis by radiologists using syngo.via software. Atherosclerosis in the carotid arteries was examined using a Siemens Acuson S2000 ultrasound machine equipped with a 9 L4 linear transducer according to a standardized protocol. Both the right and left carotid arteries were examined, and atherosclerotic plaques were identified in the common carotid artery, the carotid bulb, or the internal carotid artery.

### 2.5 Variables

#### 2.5.1 Exposure Variables

Insulin secretion was assessed using the Insulinogenic Index (IGI), a surrogate measure with a high correlation to gold standard methods of insulin secretion [16]. The IGI was calculated using the formula: ((insulin30—insulin0)/(glucose30—glucose0)). Because the IGI reflects the relationship between incremental insulin and glucose levels from baseline to 30 min, negative values or unpredictable values may occur if glucose levels fall or remain unchanged during this period. Such values are considered biologically implausible and have been treated as missing data [17]. In this study, seven participants who had either a negative IGI value or a division by zero were excluded from the IGI analyses but retained for other analyses.

Insulin sensitivity was assessed using the GUTT index, which was calculated according to the formula: ([75,000 + (glucose0—glucose120) × 0.19 × body weight]/(120 × log [(insulin0 + insulin120)/2] × [(glucose0 + glucose120)/2])) [18]. The GUTT index correlates well with gold‐standard measures of insulin sensitivity [19]. Although some study participants had extremely high GUTT index values, these were based on valid insulin and glucose values and were therefore included in the analyses.

Insulin clearance was estimated from fasting levels of insulin and C‐peptide using the following formula: Insulin clearance = (C‐peptide0/insulin0) [20]. Although there is no gold standard method for measuring insulin clearance, the fasting ratio is considered a reliable approximation of endogenous insulin clearance at steady state [21].

#### 2.5.2 Outcome Variables

CAC images were obtained using non‐contrast CT scans. Lesions were identified if they exceeded the calcium threshold of 130 Hounsfield units over at least three contiguous pixels within a volume of 1 mm^3^. The area of calcification was then multiplied by an intensity factor to derive the calcium score [15]. The calcium content in each coronary artery was summed to obtain the CACS based on the Agatston method [22]. CACS was categorized into the following groups: 0; 1–10 (ultra‐low); 11–100 (low); 101–400 (moderate); and > 400 (high) [15].

The extent of coronary atherosclerosis was also assessed using the SIS, which was determined by summing the total number of coronary segments with atherosclerotic plaques, regardless of the degree of stenosis. The SIS ranges from 0 to 18, and an SIS of more than 4 has been associated with worse cardiovascular outcomes [23, 24].

Carotid atherosclerosis was assessed by quantifying the total number of plaques in the right and left carotid arteries. According to the Mannheim consensus, plaques were defined as focal structures that protrude at least 0.5 mm or 50% of the surrounding intima‐media thickness or have a total thickness of more than 1.5 mm, measured from the media‐adventitia border to the intima‐lumen border [25].

#### 2.5.3 Covariates

Established risk factors for CVD, age, sex, LDL cholesterol, smoking status, hypertension, and body mass index (BMI) were used as covariates. Participants were classified as hypertensive if they had a systolic blood pressure > 140 mmHg, a diastolic blood pressure > 90 mmHg, or self‐reported use of antihypertensive medication. Smoking status was reported as smoker or non‐smoker, regardless of smoking history. LDL cholesterol was calculated using the Friedewald formula (LDL cholesterol = total cholesterol − HDL cholesterol − 0.45 × triglyceride level). To calculate BMI, body weight was measured on a scale and height was measured according to standardized protocols [14].

### 2.6 Statistics and Data Analysis

Association between the three exposures (insulin sensitivity, secretion, and clearance) and each of the three outcomes (CACS, SIS, and carotid plaque count) was evaluated using ordinal proportional odds regression models with a logit link function, resulting in nine different models. Ordinal regression was chosen because all three outcomes were on a discrete, non‐normally distributed scale. To account for the nonlinearity of the associations, exposure variables were modeled using restricted cubic splines [26], with knots placed at the 10th, 50th, and 90th percentiles of the distributions. The *p*‐values for the overall effect of all exposure spline terms were calculated using likelihood ratio tests. All models were adjusted for age, sex, LDL cholesterol, smoking status, hypertension, and BMI. BMI was excluded from models involving the GUTT index, as body weight is already included in the GUTT formula. Restricted cubic splines were also used for continuous covariates (age, LDL levels, and BMI).

The proportional odds assumption was evaluated by dichotomizing the outcomes at various cut‐off values and assessing the consistency of the odds ratio estimated from the corresponding binary logistic regression models.

Independent t‐tests were used to assess sex differences in insulin clearance and to compare insulin clearance between individuals with and without atherosclerosis among those with high insulin sensitivity.

For the primary analyses, missing data were handled using multiple imputations in SPSS (IBM SPSS Statistics, version 28.0.1.1), generating 30 imputed data sets. The imputations were performed using partial mean matching with five potential donors. Analyses were performed for each of the 30 data sets, and the results were then pooled using Rubin's rule. Imputations and t‐tests were performed in SPSS, while the association models were fitted in R (R version 4.4.2) using the R‐package rms [27].

## 3 Results

**FIGURE 1:**
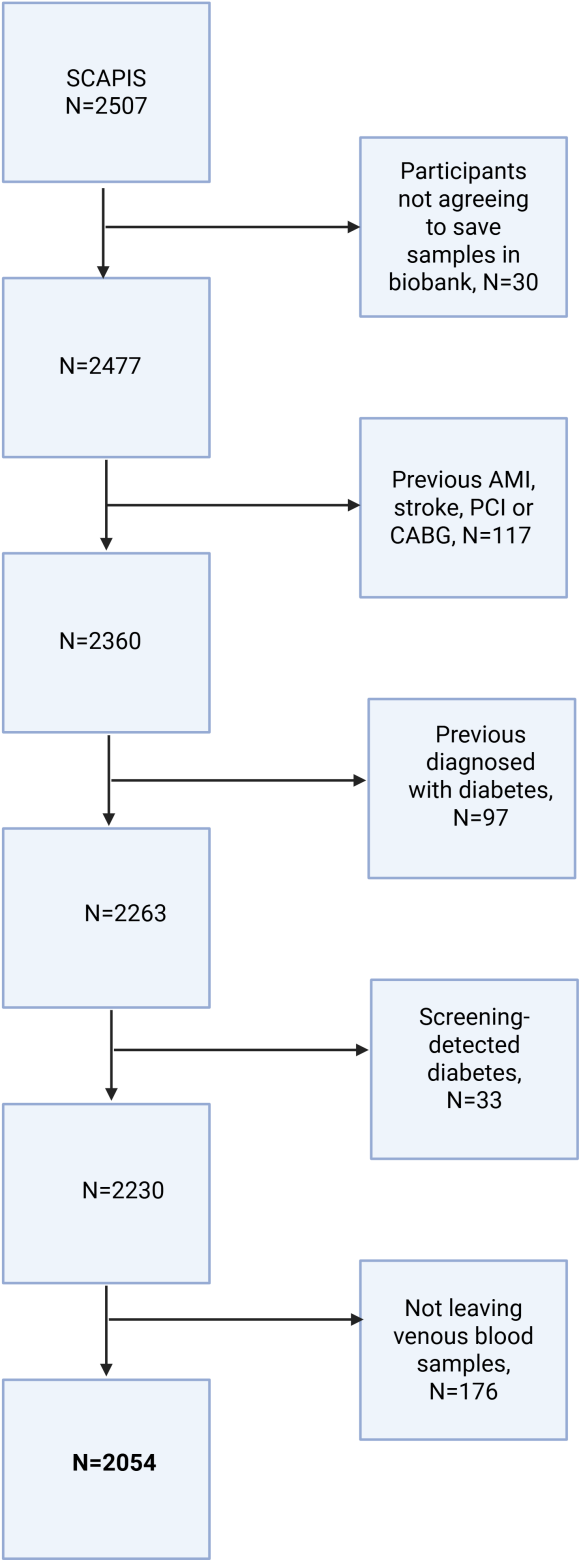
Flowchart of participant inclusion and causes for exclusion.

A total of 2507 participants were recruited for this study (Figure 1). Of these, we excluded 117 participants with clinically manifest atherosclerotic disease (defined as previous stroke, AMI, PCI, or CABG); 97 individuals with known diabetes; 33 participants with diabetes diagnosed during the study; 30 individuals who had not consented to the storage of biobank samples; and 176 individuals who had not provided blood samples for subsequent insulin analysis. This resulted in a final cohort of 2054 individuals for the analyses.

Baseline characteristics of the study cohort are presented in Table 1. The cohort was 53% female and had a mean BMI of 26.8 kg/m^2^. Approximately 22% of participants had impaired glucose regulation based on fasting glucose, 120‐min glucose, or HbA1c measurements. More than half of the cohort had no detectable signs of atherosclerosis (Table 1). The extent of missing values for each variable is provided in Table S1.

**TABLE 1:** Basic characteristics.

Characteristics	Total	Women	Men
Sample size, *n* (%)	2054 (100%)	1085 (52.8)	969 (47.2)
Age, year, mean ± SD	57.29 ± 4.31	57.44 ± 4.28	57.11 ± 4.35
Body mass Index, kg/m^2^, mean ± SD	26.82 ± 4.48	26.36 ± 4.81	27.35 ± 4.02
Smoking status, *n* (%)	1954	1041	913
Yes	161 (8.2)	91 (8.7)	70 (7.7)
No	1793 (91.8)	950 (91.3)	843 (92.3)
Hypertension, *n* (%)	2054	1085	969
Yes	790 (38.5)	382 (35.2)	408 (42.1)
No	1264 (61.5)	703 (64.8)	561 (57.9)
Glucose and insulin measures, mean ± SD			
Fasting blood sugar, mmol/L	5.16 ± 0.63	5.050 ± 0.62	5.283 ± 0.62
HbA1c, mmol/mol	37.41 ± 3.54	37.45 ± 3.60	37.36 ± 3.48
Insulin secretion	21.52 ± 25.54	21.02 ± 30.50	22.05 ± 19.02
Insulin sensitivity	86.98 ± 33.61	84.69 ± 30.81	89.33 ± 36.15
Insulin clearance	0.087 ± 0.035	0.090 ± 0.039	0.082 ± 0.028
Glycemic status, *n* (%)	1794	945	849
NGT	1392 (77.6)	741 (78.4)	651 (76.7)
IFG	120 (6.7)	43 (4.6)	77 (9.1)
IGT	103 (5.7)	65 (6.9)	38 (4.5)
IGT + IFG	34 (1.9)	12 (1.3)	22 (2.6)
Elevated HbA1c	145 (8.1)	84 (8.9)	61 (7.2)
Blood pressure, mean ± SD			
Systolic, mm Hg	125.7 ± 15.8	122.8 ± 16.1	129.0 ± 14.8
Diastolic, mm Hg	79.3 ± 9.4	78.6 ± 9.6	80.2 ± 9.2
Clinical Chemistry, mean ± SD			
Total cholesterol mmol/L	5.60 ± 1.01	5.74 ± 0.99	5.44 ± 1.01
HDL cholesterol, mmol/L	1.66 ± 0.48	1.86 ± 0.48	1.43 ± 0.38
LDL cholesterol, mmol/L	3.36 ± 0.92	3.36 ± 0.93	3.37 ± 0.91
Triglycerides, mmol/L	1.27 ± 0.67	1.15 ± 0.56	1.42 ± 0.75
SIS, *n* (%)	1917	988	929
0	1140 (59.5)	686 (69.4)	454 (48.9)
1	261 (13.6)	141 (14.3)	120 (12.9)
2	175 (891)	69 (7.0)	106 (11.4)
3	99 (5.2)	39 (3.9)	60 (6.5)
4	81 (4.2)	25 (2.5)	56 (6.0)
5	50 (2.6)	14 (1.4)	36 (3.8)
6	33 (1.7)	3 (0.3)	30 (3.2)
7	43 (2.2)	5 (0.5)	38 (4.1)
8	19 (1.0)	3 (0.3)	16 (1.7)
9	10 (0.5)	3 (0.3)	7 (0.8)
10	4 (0.2)	0 (0.0)	4 (0.4)
11	2 (0.1)	0 (0.0)	2 (0.2)
CACS, *n* (%) per group according to Agatston	2022	1064	958
0 (CACS 0)	1189 (58.8)	744 (69.9)	445 (46.5)
1 (CACS 1–10)	251 (12.4)	119 (11.2)	132 (13.8)
2 (CACS 11–100)	350 (17.3)	146 (13.7)	204 (21.3)
3 (CACS 101–400)	157 (7.7)	43 (4.0)	114 (11.9)
4 (CACS > 400)	75 (3.7)	12 (1.1)	63 (6.8)
Number of carotid plaques, *n* (%)	2049	1083	966
0	1085 (53.0)	645 (59.6)	440 (45.5)
1	484 (23.6)	243 (22.4)	241 (24.9)
2	300 (14.6)	127 (11.7)	173 (17.9)
3	129 (6.3)	51 (4.7)	78 (8.0)
4	36 (1.8)	12 (1.1)	24 (2.5)
5	12 (0.6)	4 (0.4)	8 (0.8)
6	2 (0.1)	0 (0.0)	2 (0.2)
7	1 (0.05)	1 (0.1)	0 (0.0)

*Note:* NGT, normal glucose tolerance; IGT, impaired glucose tolerance (2‐h glucose level of 7.8–11.0 mmol/L following an oral glucose tolerance test); IFG, Impaired fasting glucose (fasting glucose level at 6.1–6.9 mmol/L); IGT + IFG, combined impaired fasting and post‐load glucose levels; Elevated HbA1c, HbA1c between 42 and 48 mmol/mol.

### 3.1 Insulin Sensitivity

**FIGURE 2:**
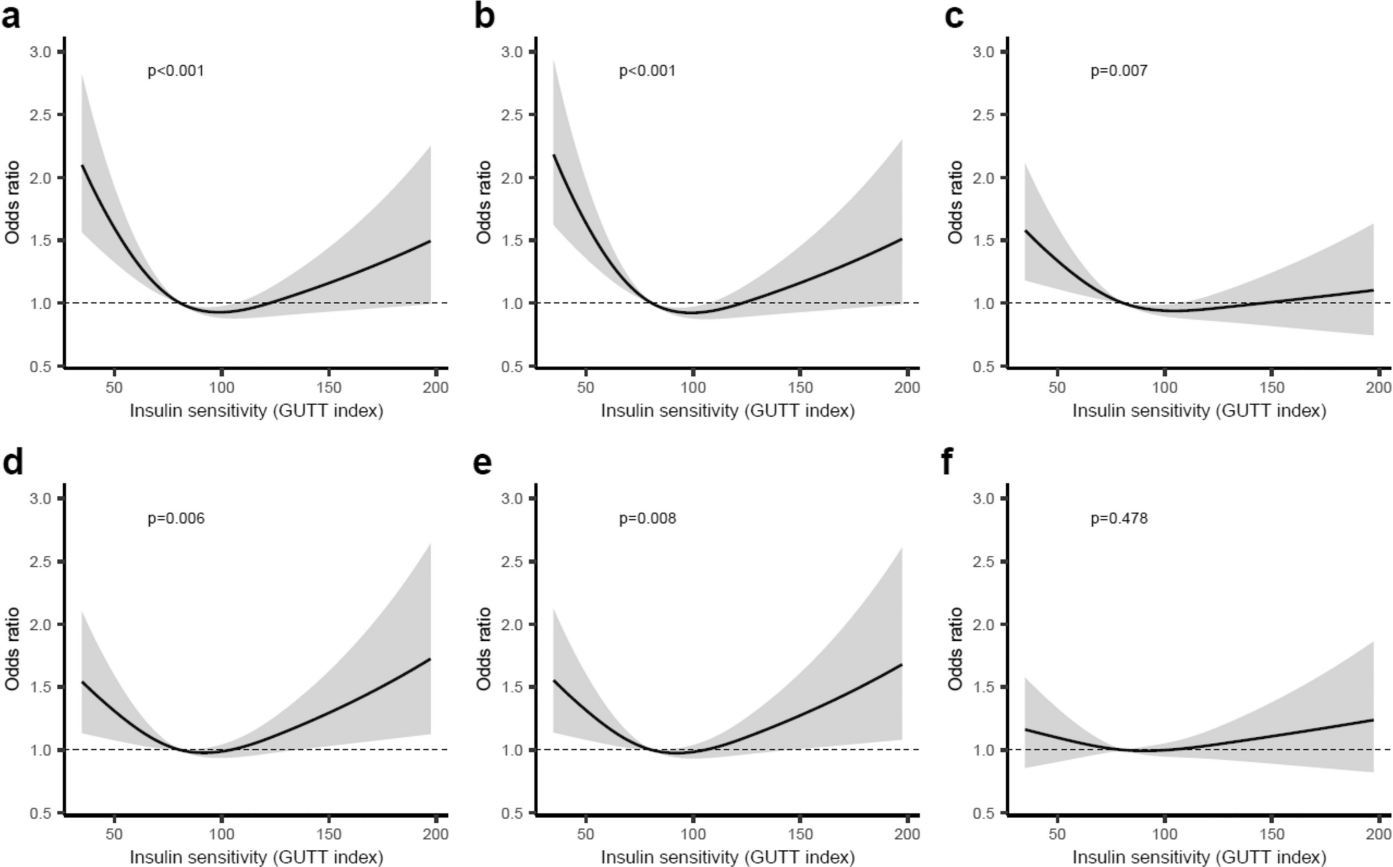
Associations between insulin sensitivity and measures of subclinical atherosclerosis. Panels a‐c show unadjusted (crude) associations between insulin sensitivity (assessed by the GUTT index) and (a) Segment Involvement Score (SIS), (b) Coronary artery calcium score (CACS), and (c) number of carotid plaques. Panels (d–f) show the corresponding associations after adjustment for age, sex, low‐density lipoprotein (LDL) cholesterol, smoking status and hypertension: (d) SIS, (e) CACS and (f) number of carotid plaques. All figures show predicted odds ratios with 95% confidence interval bands, using the median value of the insulin sensitivity measure as the reference point.

Associations between insulin sensitivity, assessed using the GUTT index, and the three atherosclerosis measures are shown in Figure 2. In unadjusted models, lower insulin sensitivity was significantly associated with higher SIS (*p* < 0.001, Figure 2a), higher CACS (*p* < 0.001, Figure 2b), and a greater number of carotid plaques (*p* = 0.007, Figure 2c). After adjustment for potential confounders, the association remained significant for SIS (*p* = 0.006, Figure 2d) and CACS (*p* = 0.008, Figure 2e), but not for carotid plaque count (*p* = 0.478, Figure 2f).

A U‐shaped relationship was observed between insulin sensitivity and both SIS and CACS, indicating that both low and high levels of insulin sensitivity were associated with atherosclerosis. As this pattern was unexpected, further investigations were performed using scatter plots of SIS, CACS, and the number of plaques in the carotid arteries against insulin sensitivity (Figure S1). Fourteen people were found to have extremely high insulin sensitivity, with GUTT values exceeding 200. These participants had low glucose and insulin levels both at baseline and after 120 min, with peak values observed at 30 min—a time point not captured by the GUTT calculation. Of these 14 subjects with high GUTT values, seven showed signs of atherosclerosis. When these seven subjects with atherosclerosis were compared with the seven subjects without atherosclerosis, no differences were observed in LDL levels, BMI, age, sex, hypertension, or smoking status (Table S2). The observed U‐shaped relationship was not substantially modified by the exclusion of these individuals (Figure S2a–c), and they were retained in the primary analysis.

### 3.2 Insulin Clearance

**FIGURE 3:**
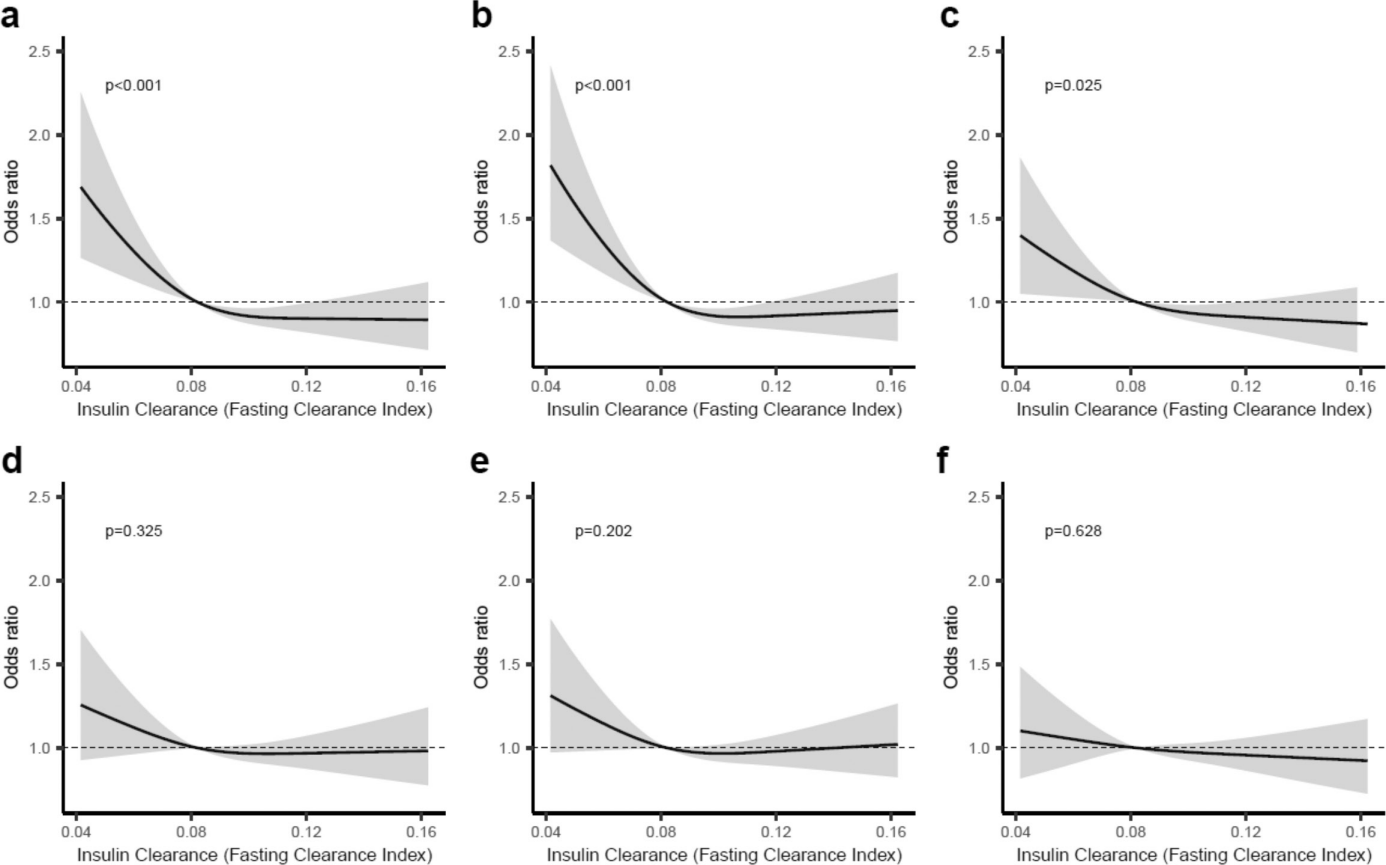
Associations between insulin clearance and measures of subclinical atherosclerosis. Panels a‐c display unadjusted (crude) associations between insulin clearance (assessed by fasting clearance index) and (a) Segment Involvement score (SIS), (b) Coronary artery calcium score (CACS) and (c) number of carotid plaques. Panels d‐f show the corresponding associations after adjustment for age, sex, low‐density lipoprotein (LDL) cholesterol, smoking status and hypertension: (d) SIS, (e) CACS and (f) number of carotid plaques. All figures show predicted odds ratios with 95% confidence interval bands, using the median value of the insulin clearance measure as the reference point.

The relationship between insulin clearance, estimated by the fasting C‐peptide to insulin ratio, and atherosclerosis is shown in Figure 3. In unadjusted models, lower insulin clearance was significantly associated with higher SIS (*p* < 0.001, Figure 3a), higher CACS (*p* < 0.001, Figure 3b), and a greater number of carotid plaques (*p* = 0.025, Figure 3c). However, these associations were attenuated and no longer statistically significant after adjustment for confounders (Figure 3d–f).

Of note, women had significantly higher insulin clearance than men (*p* < 0.001). We progressively expanded sets of covariates to further investigate the specific effects of individual confounders. The associations of insulin clearance with SIS and CACS remained statistically significant after adjustment for age alone (Figure S3a–c), but only with CACS after adjustment for age and sex (Figure S3d–f) and were no longer significant after further adjustment for hypertension and smoking status (Figure S3g–i).

### 3.3 Insulin Secretion

**FIGURE 4:**
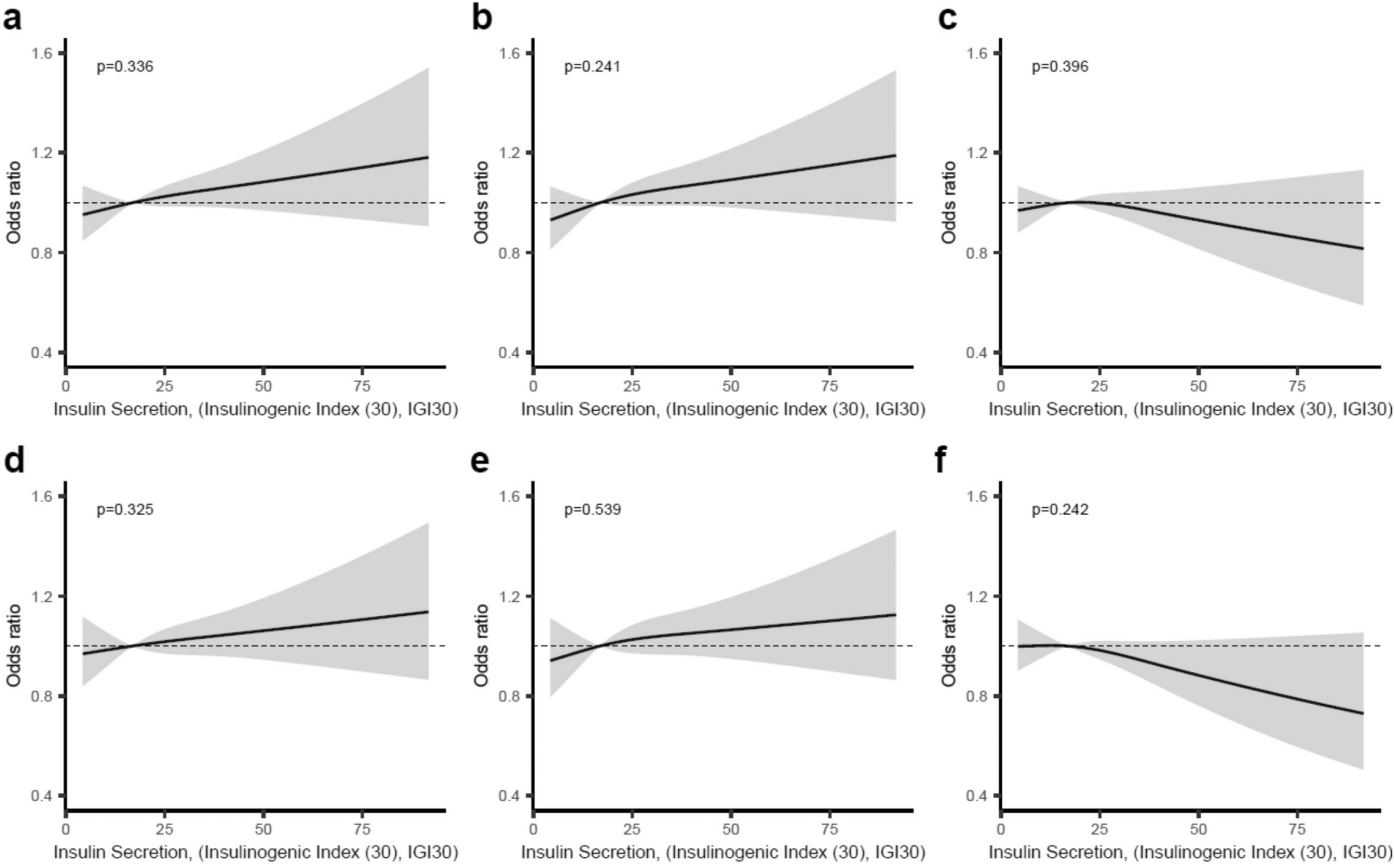
Associations between insulin secretion and measures of subclinical atherosclerosis. Panels a‐c show unadjusted (crude) associations between insulin secretion (assessed by the Insulinogenic index) and (a) Segment Involvement Score (SIS), (b) Coronary artery calcium score (CACS), and (c) number of carotid plaques. Panels (d–f) show the corresponding associations after adjustment for age, sex, low‐density lipoprotein (LDL) cholesterol, smoking status and hypertension: (d) SIS, (e) CACS and (f) number of carotid plaques. All figures show predicted odds ratios with 95% confidence interval bands, using the median value of the insulin secretion measure as the reference point.

No significant associations were observed between insulin secretion, assessed with the IGI, and any of the three atherosclerotic measures, either in unadjusted (Figure 4a–c) or adjusted models (Figure 4d–f).

### 3.4 Sensitivity Analysis

In the analyses using the original data without imputation, the results were consistent with those of the imputed models. The number of participants included in each model is shown in Table S3, and missing data by variable are detailed in Table S1. Models using the original data are presented in Figures S4–S6.

## 4 Discussion

In this population‐based study of individuals without diabetes or clinically manifest atherosclerosis, we investigated the association between insulin sensitivity, secretion, and clearance with subclinical atherosclerosis. We found that lower insulin sensitivity was significantly associated with increased CACS and SIS, both established predictors of atherosclerotic disease. In contrast, no associations were found between insulin secretion and any of the atherosclerotic measures, and the associations between insulin clearance and subclinical atherosclerosis were no longer observed after adjustment for potential confounders.

Our results emphasize the central role of insulin resistance in the early stages of atherosclerosis development. This is consistent with previous studies suggesting that insulin promotes atherosclerosis through multiple mechanisms [28, 29]. These include impaired activation of the PI3K‐AKT pathway, which leads to reduced glucose uptake and diminished activation of antiatherogenic factors such as nitric oxide [6]. At the same time, insulin resistance can activate the mitogen‐activated protein kinase (MAPK) pathway, which promotes inflammation and smooth muscle cell proliferation and further increases insulin resistance [30]. In addition, insulin resistance leads to hyperinsulinemia, which can promote atherogenesis through mechanisms such as increased LDL synthesis, increased vascular inflammation, weight gain, and hypertension due to sodium retention [6, 31, 32].

An unexpected finding was the U‐shaped relationship between insulin sensitivity and coronary atherosclerosis, where both low and high insulin sensitivity were associated with increased SIS and CACS. This pattern could not be explained by the presence of conventional CVD risk factors in participants with exceptionally high insulin sensitivity. Potential explanations include limitations of the surrogate measure used (GUTT index) or reflect underlying genetic predispositions that simultaneously influence insulin dynamics and atherosclerosis risk. Further studies are needed to elucidate the mechanisms underlying this observation.

We demonstrated a relationship between insulin sensitivity and atherosclerosis in the coronary arteries, but not in the carotid arteries after adjustment for CVD risk factors. CACS and SIS are well‐established predictors of atherosclerotic disease in the coronary arteries, whereas carotid artery plaque burden is more closely related to cerebrovascular events [33, 34, 35]. The observed difference may reflect site‐specific pathophysiological mechanisms, or it could be due to limitations in the sensitivity of the carotid plaque measurement used [36]. Further research is warranted to investigate the differential impact of insulin resistance on various vascular beds.

We found no association between insulin secretion and subclinical atherosclerosis. It is possible that the increased risk of atherosclerosis is primarily related to insulin resistance and that insulin secretion itself has only a limited direct effect on the atherosclerotic process. Another possibility is that the surrogate measure used in this study, the IGI, which only reflects insulin secretion in the early phase, may be an inadequate measure to capture relevant aspects of beta cell function related to atherogenesis.

Similarly, reduced insulin clearance was associated with higher atherosclerotic burden in unadjusted models, but these associations did not persist after multivariable adjustment. This is in contrast to some previous studies suggesting that impaired hepatic insulin clearance contributes to hyperinsulinemia and elevated CVD risk [9]. In our study, the association remained after adjusting for age but was attenuated when adjusting for sex. This may reflect known sex differences in insulin clearance, with higher clearance in women [37].

A major strength of our study is the large, randomly selected population‐based sample with a high participation rate and comprehensive phenotyping. Insulin dynamics were derived from OGTT‐based measures, and subclinical atherosclerosis was assessed using state‐of‐the‐art imaging techniques. Associations were analyzed using the recommended method for nonlinearity, restricted cubic splines, to avoid losing important information in the analysis. However, our study also has limitations. These include missing OGTT data and the use of surrogate measures of insulin sensitivity, secretion, and clearance, which may not fully capture the complexity of these processes. In addition, the study population was limited to individuals between the ages of 50 and 64, primarily of European descent, which limits the generalizability of the results to other age groups or ethnicities. Most importantly, this study is a cross‐sectional study. A longitudinal study would be required to investigate causality.

### 4.1 Implications

Since increased insulin resistance in non‐diabetic individuals is associated with subclinical atherosclerosis, identifying individuals with increased insulin resistance and taking preventive measures against the progression of atherosclerosis can reduce the risk of CVD. Insulin resistance is a modifiable risk factor for atherosclerosis that can be addressed through lifestyle interventions, such as increased physical activity, dietary changes, and weight loss [38]. Future clinical trials may clarify the potential role of pharmacological interventions preventing metabolic deterioration and halting the progression of atherosclerosis [38].

In conclusion, our study reinforces the central role of insulin resistance in the development of coronary atherosclerosis, even in individuals without diabetes. Neither insulin secretion nor clearance showed an association with atherosclerosis after adjustment for established CVD risk factors. Longitudinal studies are needed to clarify causal relationships and to explore the interplay between insulin sensitivity, secretion, clearance, and genetic or environmental factors in the pathogenesis of atherosclerosis and CVD.

## Author Contributions

R.R., T.O., and J.O. designed the study. P.L. performed the statistical analyses together with J.O. and R.R. R.R. wrote the first draft of the manuscript. J.O. wrote, reviewed, and edited the manuscript together with I.K., P.L., and T.O. T.O., P.L., I.K., and J.O. also participated in the discussion. All authors have read and approved the published version of the manuscript. RR is the guarantor of this work.

## Ethics Statement

The study was approved by the Swedish Ethical Review Board (DNR2010‐228‐31M) for the baseline examination included in SCAPIS and by the Swedish Ethical Review Board in Umeå, Sweden (Dnr 2016‐151‐31) for the OGTT‐substudy.

## Conflicts of Interest

The authors declare no conflicts of interest.

## Supporting information


**Data S1:** Supporting Information.

## Data Availability

The data sets analyzed during the current study are available from the corresponding author on reasonable request.
