# Supplementary material for: The Association of Insulin Sensitivity, Secretion, and Clearance With Subclinical Atherosclerosis in Middle‐Aged Adults Without Diabetes: A Cross‐Sectional Analysis of the SCAPIS Cohort
Source: J Diabetes. 2025 Oct 19;17(10):e70161. doi: 10.1111/1753-0407.70161 (PMC12536067; doi:10.1111/1753-0407.70161)

Supplementary Table 1: Missing data in participants included in the analyses (n=2054).

| Characteristic | Total |
| --- | --- |
| Age— n (%) | 0 (0.0) |
| Body mass index — n (%) | 0 (0.0) |
| Smoking status (yes/no) — n (%) | 73 (3.6) |
| Antihypertensive medication — n (%) | 0 (0.0) |
| Systolic blood pressure — n (%) | 1 (0.0) |
| Diastolic blood pressure — n (%) | 1 (0.0) |
| Total cholesterol — n (%) | 0 (0.0) |
| HDL cholesterol — n (%) | 0 (0.0) |
| LDL cholesterol — n (%) | 17 (0.8) |
| Triglycerides — n (%) | 0 (0.0) |
| Glucose fasting— n (%) | 4 (0.2) |
| Glucose 30 minutes— n (%) | 277 (13.5) |
| Glucose 120 minutes— n (%) | 273 (13.3) |
| Insulin fasting— n (%) | 0 (0.0) |
| Insulin 30 minutes— n (%) | 407 (19.8) |
| Insulin 120 minutes— n (%) | 438 (21.3) |
| C-peptide fasting— n (%) | 28 (1.4) |
| HbA1c — n (%) | 7 (0.3) |
| Insulin secretion | 409 (19.9) |
| Insulin sensitivity | 423 (20.6) |
| Insulin clearance | 26 (1.3) |
| SIS | 137 (6.7) |
| Number of carotid plaques | 5 (0.2) |
| CACS | 32 (1.6) |

Supplementary figure 1. Scatter plots of Segment Involvement score, SIS, (A), Coronary artery calcium score, CACS, (B) and number of plaques in the carotids (C) by insulin sensitivity.

| 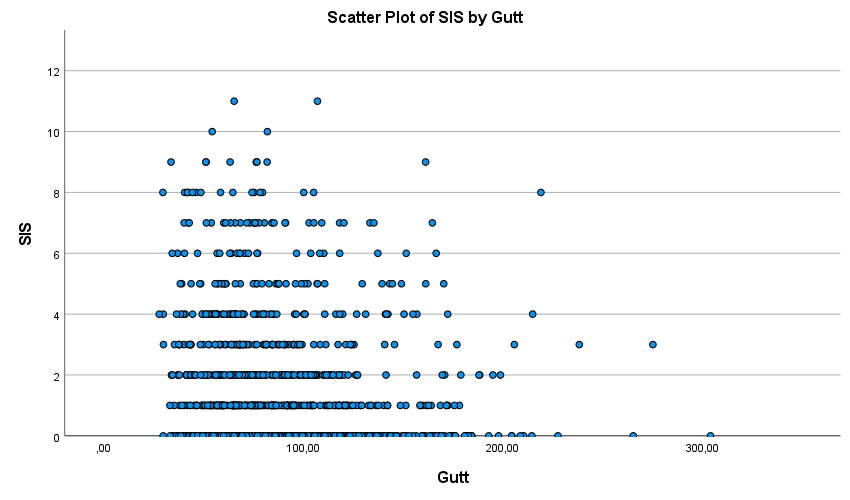 | 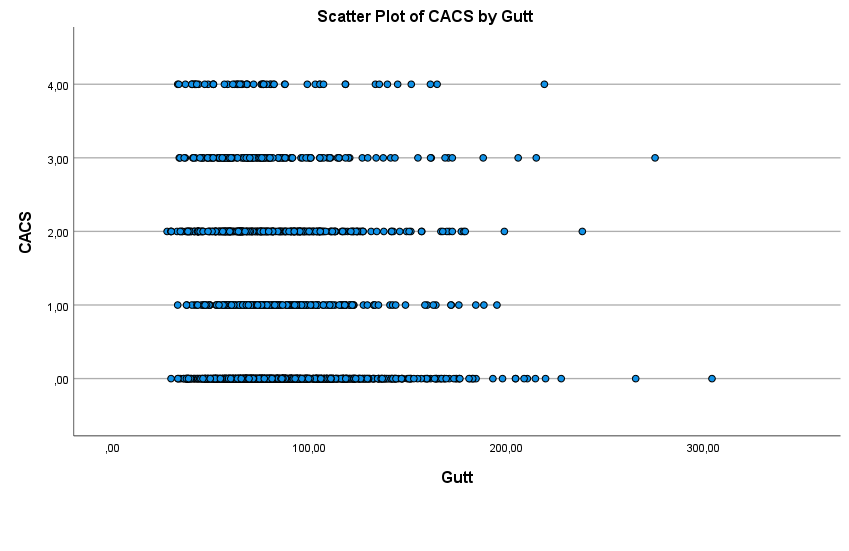 | 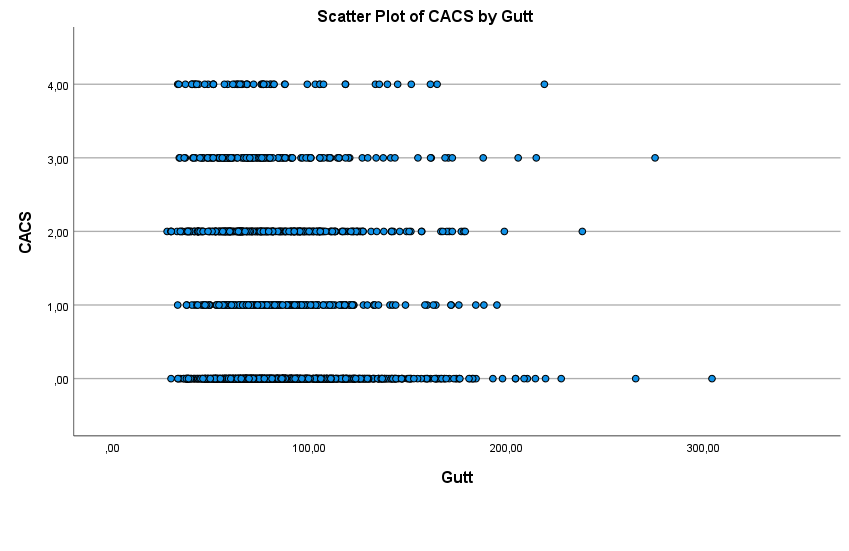 |
| --- | --- | --- |
| Scatter plot Segment Involvement score (SIS) by Insulin sensitivity. | Scatter plot of Coronary artery calcium score (CACS) by Insulin sensitivity. | Scatter plot of the number of plaques in carotid arteries by Insulin sensitivity. |

Supplementary table 2. Characteristics of participants with extremely high insulin sensitivity (GUTT above 200). Comparison of individuals with and without atherosclerosis (SIS, CACS or carotid plaques).

|  | **Participants with GUTT above 200** | |  | **All study participants** |
| --- | --- | --- | --- | --- |
|  | **With atherosclerosis** | **Without atherosclerosis** | **Difference between participants with and without atherosclerosis** |  |
| n | 7 | 7 |  | 2054 |
| Women, n (%) | 3 (43) | 2 (29) | p=0.710 | 1085 (53) |
| Age, y (mean ± SD) | 55.43 ± 4.44 | 56.11 ± 5.08 | p=0.971 | 57.29 ± 4.31 |
| BMI (mean ± SD) | 25.56 ± 1.44 | 23.66 ± 2.47 | p=0.198 | 26.82 ± 4.48 |
| Hypertension, n (%) | 4 (57) | 1 (14) | p=0.209 | 790 (38%) |
| Smoking, n (%) | 0 (0) | 0 (0) | p=1.00 | 161 (8.2) |
| LDL (mean ± SD) | 3.13 ± 0.96 | 3.0 ± 0.71 | p=0.183 | 3.36 ± 0.92 |
| Insulin sensitivity (mean ± SD) | 239.0 ± 37.0 | 220.3 ± 21.7 | p=0.155 | 87.0 ± 33.6 |
| SIS, n (%) | 3 (43) | 0 | p<0.05 | - |
| CACS, n (%) | 3 (43) | 0 | p<0.05 | - |
| Plaques in carotid arteries, n (%) | 1 (14) | 0 | p<0.05 | - |

Supplementary figure 2. Associations between insulin sensitivity and measures of subclinical atherosclerosis. Panels a-c show unadjusted (crude) associations between insulin sensitivity (assessed by the GUTT index) and a) Segment Involvement Score (SIS), b) Coronary artery calcium score (CACS), and c) number of carotid plaques. Individuals with extreme values, GUTT above 200, are excluded.
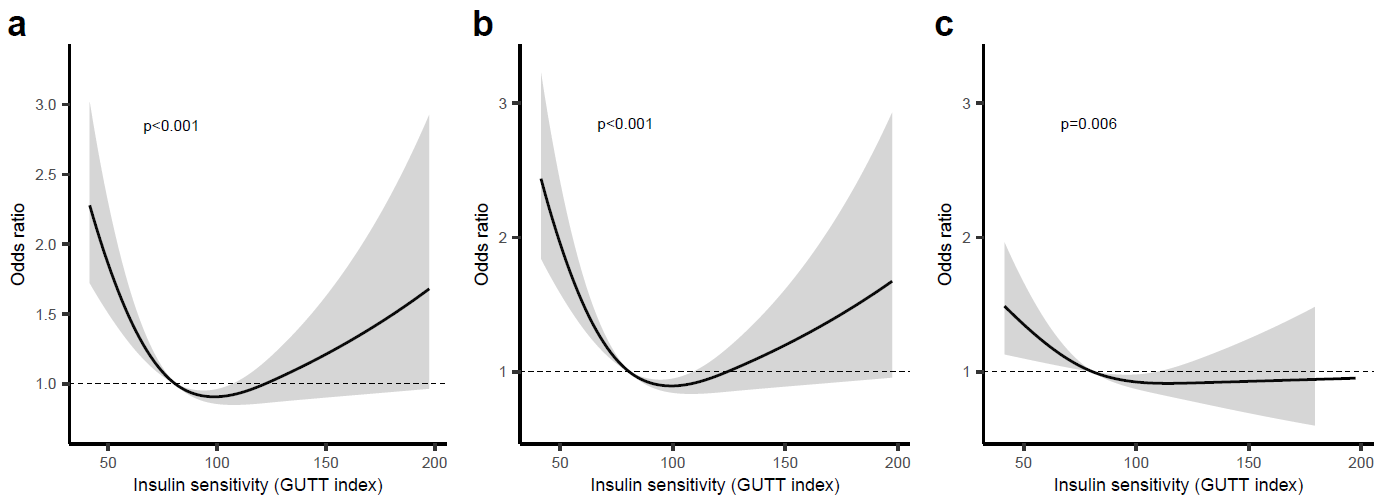


Supplementary figure 3. Associations between insulin clearance and measures of subclinical atherosclerosis. Panels a-c display associations adjusted for age between insulin clearance (assessed by fasting clearance index) and a) Segment Involvement score (SIS), b) Coronary artery calcium score (CACS) and c) number of carotid plaques. Panels d-f show the corresponding associations after adjustment for age and sex: d) SIS, e) CACS and f) number of carotid plaques. Panels g-i show the corresponding associations after adjustment for age, sex, hypertension and smoking: g) SIS, h) CACS and i) number of carotid plaques. All figures show predicted odds ratios with 95% confidence interval bands, using the median value of the insulin clearance measure as the reference point.


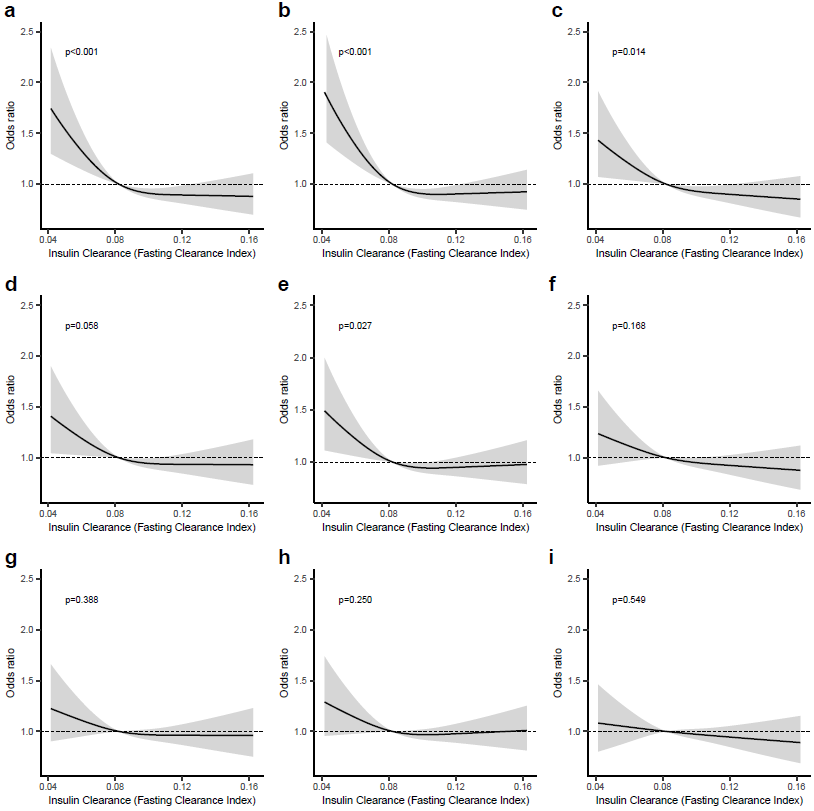


Supplementary table 3. Number of participants included in each model with confounders, original data. (SIS= Segment Involvement score; CACS= Coronary artery calcium score; LDL= low density lipoprotein)

| **Exposure** | **Outcome** | **Confounder** | **N** |
| --- | --- | --- | --- |
| Insulin sensitivity | SIS | - | 1533 |
| Insulin sensitivity | SIS | Age | 1533 |
| Insulin sensitivity | SIS | Age and sex | 1533 |
| Insulin sensitivity | SIS | Age, sex, hypertension and smoking | 1488 |
| Insulin sensitivity | SIS | Age, sex, hypertension, smoking and LDL | 1474 |
| Insulin sensitivity | CACS | - | 1600 |
| Insulin sensitivity | CACS | Age | 1600 |
| Insulin sensitivity | CACS | Age and sex | 1600 |
| Insulin sensitivity | CACS | Age, sex, hypertension and smoking | 1554 |
| Insulin sensitivity | CACS | Age, sex, hypertension, smoking and LDL | 1539 |
| Insulin sensitivity | Number of plaques in the carotid arteries | - | 1618 |
| Insulin sensitivity | Number of plaques in the carotid arteries | Age | 1618 |
| Insulin sensitivity | Number of plaques in the carotid arteries | Age and sex | 1618 |
| Insulin sensitivity | Number of plaques in the carotid arteries | Age, sex, hypertension and smoking | 1571 |
| Insulin sensitivity | Number of plaques in the carotid arteries | Age, sex, hypertension, smoking and LDL | 1556 |
| Insulin clearance | SIS | - | 1896 |
| Insulin clearance | SIS | Age | 1896 |
| Insulin clearance | SIS | Age and sex | 1896 |
| Insulin clearance | SIS | Age, sex, hypertension and smoking | 1832 |
| Insulin clearance | SIS | Age, sex, hypertension, smoking and LDL | 1817 |
| Insulin clearance | CACS | - | 1996 |
| Insulin clearance | CACS | Age | 1996 |
| Insulin clearance | CACS | Age and sex | 1996 |
| Insulin clearance | CACS | Age, sex, hypertension and smoking | 1927 |
| Insulin clearance | CACS | Age, sex, hypertension, smoking and LDL | 1911 |
| Insulin clearance | Number of plaques in the carotid arteries | - | 2023 |
| Insulin clearance | Number of plaques in the carotid arteries | Age | 2023 |
| Insulin clearance | Number of plaques in the carotid arteries | Age and sex | 2023 |
| Insulin clearance | Number of plaques in the carotid arteries | Age, sex, hypertension and smoking | 1952 |
| Insulin clearance | Number of plaques in the carotid arteries | Age, sex, hypertension, smoking and LDL | 1936 |
| Insulin secretion | SIS | - | 1550 |
| Insulin secretion | CACS | - | 1621 |
| Insulin secretion | Number of plaques in the carotid arteries | - | 1641 |

Supplementary figure 4. Original data. Associations between insulin sensitivity and measures of subclinical atherosclerosis. Panels a-c show unadjusted (crude) associations between insulin sensitivity (assessed by the GUTT index) and a) Segment Involvement Score (SIS), b) Coronary artery calcium score (CACS), and c) number of carotid plaques. Panels d-f show the corresponding associations after adjustment for age, sex, low-density lipoprotein (LDL) cholesterol, smoking status and hypertension: d) SIS, e) CACS and f) number of carotid plaques. All figures show predicted odds ratios with 95% confidence interval bands, using the median value of the insulin sensitivity measure as the reference point.


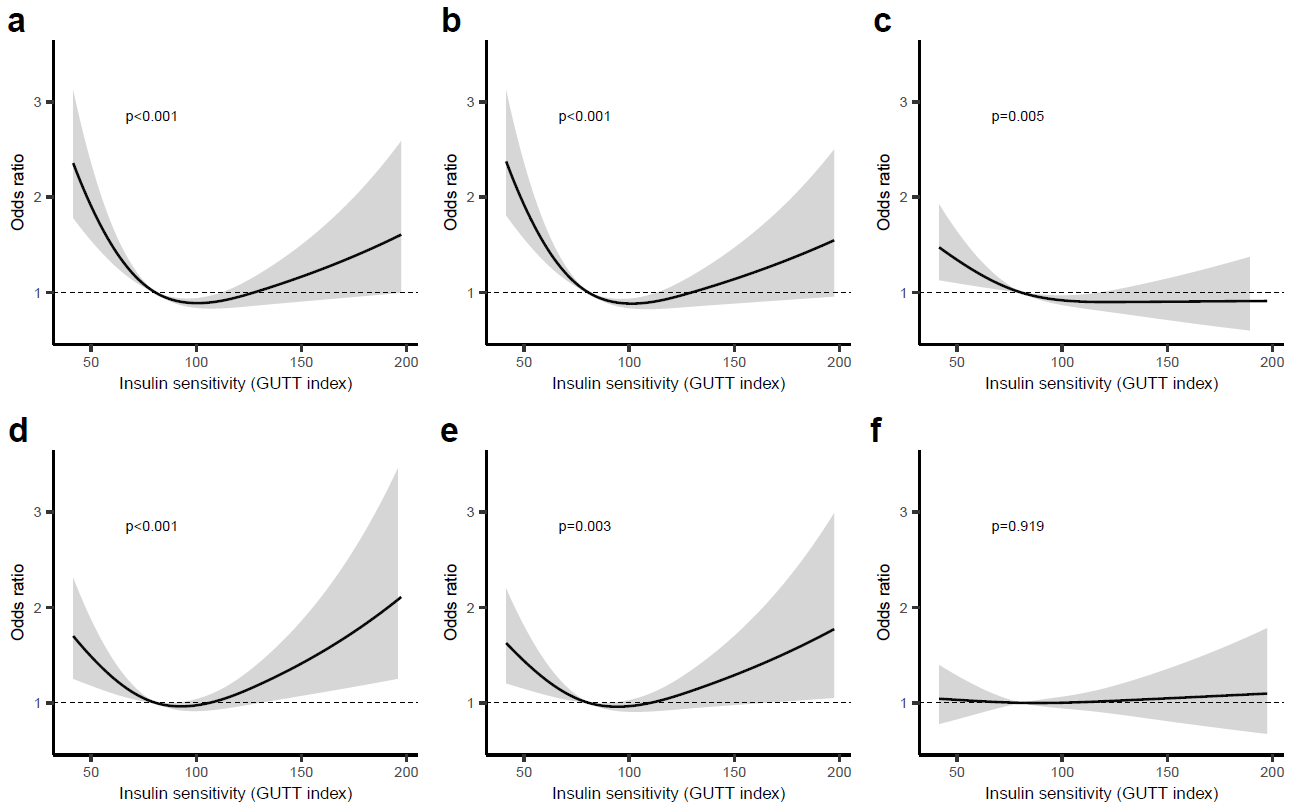


Supplementary Figure 5. Original data. Associations between insulin clearance and measures of subclinical atherosclerosis. Panels a-c display unadjusted (crude) associations between insulin clearance (assessed by fasting clearance index) and a) Segment Involvement score (SIS), b) Coronary artery calcium score (CACS) and c) number of carotid plaques. Panels d-f show the corresponding associations after adjustment for age, sex, low-density lipoprotein (LDL) cholesterol, smoking status, BMI and hypertension: d) SIS, e) CACS and f) number of carotid plaques. All figures show predicted odds ratios with 95% confidence interval bands, using the median value of the insulin clearance measure as the reference point.


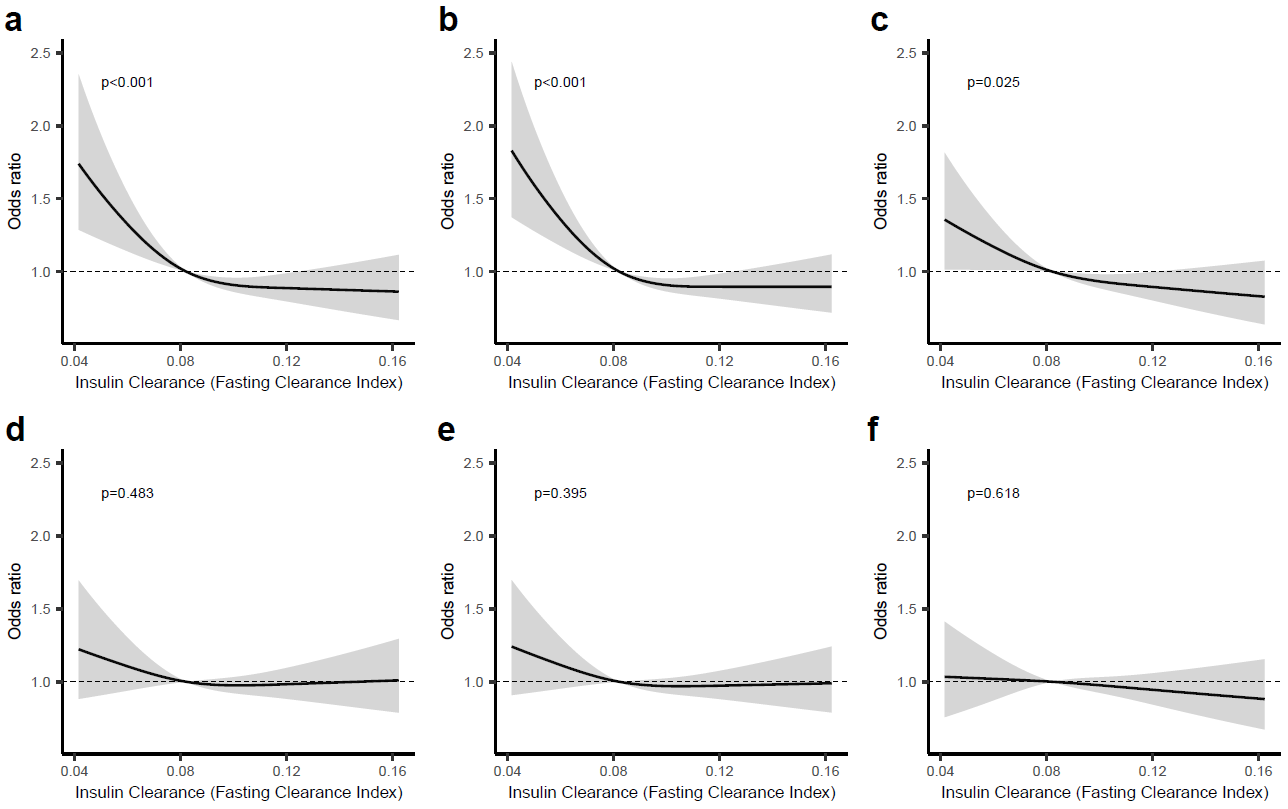


Supplementary figure 6. Original data. Associations between insulin secretion and measures of subclinical atherosclerosis. Panels a-c show unadjusted (crude) associations between insulin secretion (assessed by the Insulinogenic index) and a) Segment Involvement Score (SIS), b) Coronary artery calcium score (CACS), and c) number of carotid plaques. All figures show predicted odds ratios with 95% confidence interval bands, using the median value of the insulin secretion measure as the reference point.


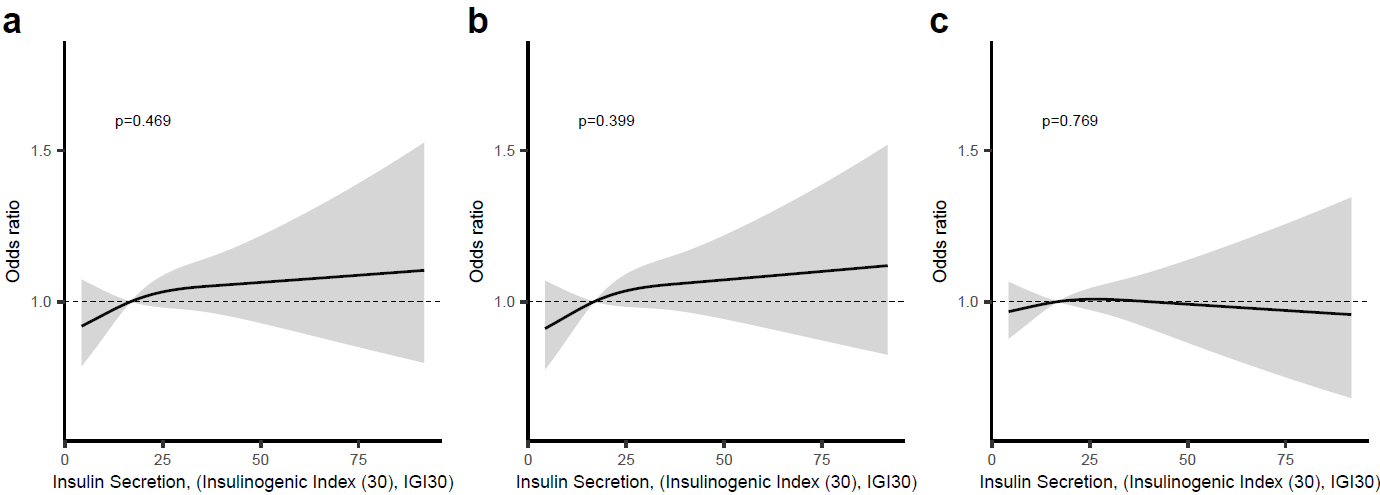

Supplement: Supplementary file 1 — Data S1: Supporting Information. [file JDB-17-e70161-s001.docx]
